# Taurolidine-containing solution for reducing cardiac implantable electronic device infection-early report from the European TauroPace™ registry

**DOI:** 10.1186/s13019-024-03059-1

**Published:** 2024-10-04

**Authors:** Reinhard Vonthein, Benito Baldauf, Stefan Borov, Ernest W. Lau, Marzia Giaccardi, Ojan Assadian, Christelle Haddad, Philippe Chévalier, Kerstin Bode, Paul Foley, Honey Thomas, Niall G. Campbell, Stephanie Fichtner, Luca Donazzan, Felix Pescoller, Rainer Oberhollenzer, Roberto Cemin, Hendrik Bonnemeier

**Affiliations:** 1 Institut für Medizinische Biometrie und Statistik, Universität zu Lübeck, Ratzeburger Allee 160, 23562 Lübeck, Germany; 2 Institute of Life Sciences, Hochschule Bremerhaven, University of Applied Sciences Bremerhaven, An der Karlstadt 8, 27568 Bremerhaven, Germany; 3 Medical Faculty, Christian-Albrechts University, Christian-Albrechts-Platz 4, 24118 Kiel, Germany; 4 Department of Cardiology, Klinikum Freising, Alois-Steinecker-Straße 18, 85354 Freising, Germany; 5 Department of Cardiology, Royal Victoria Hospital, Grosvenor Road, Belfast, BT12 6BA UK; 6 Department of Cardiology, Ospedale Santa Maria Annunziata, Ponte a Niccheri, 50012 Florence, Italy; 7 Regional Hospital Wiener Neustadt, Corvinusring 3-5, 2700 Wiener Neustadt, Austria; 8 Institute for Skin Integrity and Infection Prevention, School of Human and Health Sciences, University of Huddersfield, Huddersfield, HD1 3DH UK; 9 Department of Cardiology, Hôpital Louis Pradel, 59 Bd Pinel, 69500 Bron, France; 10 Department of Electropyhsiology, Herzzentrum Leipzig, Strümpellstraße 39, 04289 Leipzig, Germany; 11 Department of Cardiology, Great Western NHS Foundation Trust, Marlborough Rd, Swindon, SN3 6BB UK; 12 Department of Cardiology, Northumbria Healthcare NHS Foundation Trust, Ashington, Northumberland NE63 9JJ UK; 13 Division of Cardiovascular Sciences, School of Medical Sciences, Manchester Academic Health Science Centre, University of Manchester, Manchester, UK; 14 Department of Cardiology, Krankenhaus Landshut Achdorf, Achdorfer Weg 5, 84036 Landshut, Germany; 15 Department of Cardiology, Ospedale Regionale San Maurizio, Bolzano, Via Lorenz Böhler 5, 39100 Bolzano, Italy

**Keywords:** Surgical site infection, Antimicrobial compound, Permanent pacemaker, Subcutaneous implantable cardioverter defibrillator, Cardiac resynchronization therapy, Cardiac contractility modulation, Taurolidine

## Abstract

**Introduction:**

Infection is a significant complication of cardiac implantable electronic device (CIED) therapy. The European TauroPace™ Registry investigates the safety and efficacy of TauroPace™ (TP), an antimicrobial solution containing taurolidine, designed to prevent CIED infections.

**Methods:**

This multicenter study included patients undergoing CIED procedures at participating centers where TP was used as a disinfectant for external hardware surfaces and an antiseptic for irrigating surgical sites. All patients eligible for CIED placement with adjunctive TP as the standard of care were included. Other aspects of CIED procedures adhered to current guidelines. Data on CIED-related infective endocarditis, CIED pocket infection, device and procedure-related complications, adverse events, and all-cause mortality were prospectively collected for 12 months. In cases of revision, the previous procedure was censored, and a new procedure was created. Binomial and Kaplan–Meier statistics were employed to analyze event rates.

**Results:**

From January 2020 to November 2022, TP was used in 822 out of 1170 CIED procedures. Among patients who completed the 3-month follow-up, no CIED pocket infections were observed, and one case of CIED-related infective endocarditis was reported. In the 12-month follow-up cohort, two additional local pocket CIED infections were observed, resulting in a total of three major CIED infections within 1 year after the CIED placement procedure. The 3-month and 12-month major CIED infection rates were 0.125% and 0.51%, respectively. During the observation a complication rate of 4.4% was reported. No adverse events related to TP were observed.

**Conclusions:**

TP appears to be effective and safe in preventing CIED infections.

*ClinicalTrials.gov Identifier*: NCT04735666.

**Supplementary Information:**

The online version contains supplementary material available at 10.1186/s13019-024-03059-1.

## Central illustration

Prevention of cardiac implantable electronic device infection with a taurolidine containing solution.
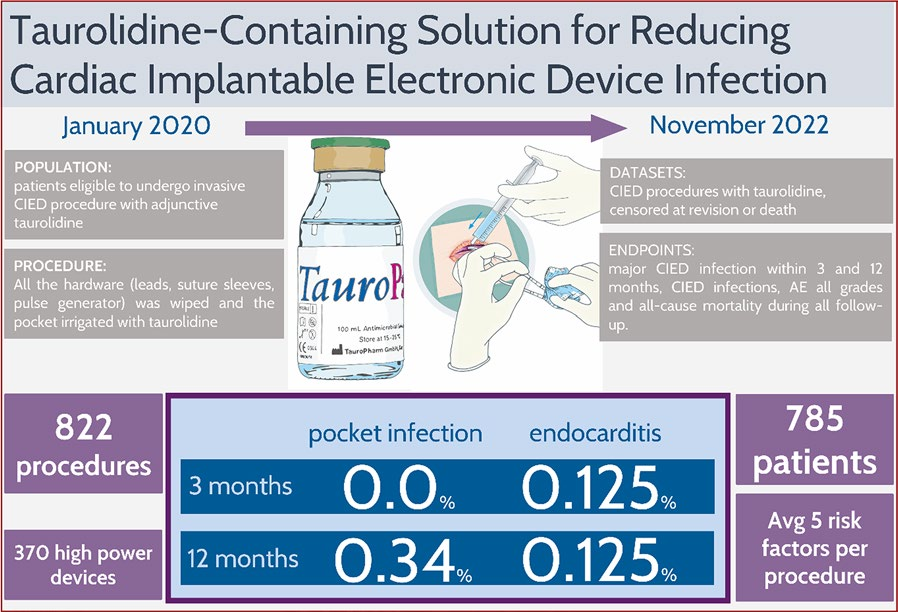


## Introduction

The implantation of cardiac implantable electronic devices (CIEDs) is an effective intervention for the treatment of arrhythmias and the prevention of sudden cardiac death [1, 2]. CIED infection represents a significant procedural complication, with infection rates increasing more rapidly than the growth in procedural volume. Contributing factors may include patient-related aspects, such as advanced age, frailty, and a higher prevalence of comorbidities, as well as procedural elements, such as the increased complexity of CIED systems and a greater proportion of revision procedures [3–13]. CIED infection is associated with substantial morbidity and both short- and long-term mortality [12, 14, 15]. It incurs substantial healthcare costs, ranging from US$77,397 to $362,606 in the USA and €59,419 in Europe [14, 16, 17].

Pre-operative antibiotic administration [18, 19] and the use of antibiotic-eluting envelopes [20–22] have been demonstrated to reduce infection rates and are recommended in contemporary guidelines [23, 24]. Novel, economically feasible approaches are required to further reduce infection rates.

TauroPace™ (TP, TauroPharm GmbH, Bavaria, Germany) is a broad-spectrum antimicrobial solution comprising taurolidine, capable of functioning as both an antiseptic and a disinfectant, and exhibits no documented resistance. The European TauroPace™ Registry (ETPR) [25] reports on the clinical safety and efficacy of TP for these applications.

In contrast to disinfectants, that may contain ethanol for rapid activity, TP stands out with its active ingredient being taurolidine, an antimicrobial compound with a clinical history dating back to the 1980’s [26–31]. The resurgence of interest in taurolidine is attributed to its established safety, efficacy, broad antimicrobial spectrum, no known microbial resistance, and versatile application [13, 25, 32–35]. Taurolidine, the active compound in TP that accounts for its broad-spectrum antimicrobial properties, is derived from the non-essential amino acid taurine. In vivo, taurolidine undergoes hydrolysis to form N-methylol taurinamide and N-methylol taurultam, which display a half-life of approximately 1 and 6 h [31, 36]. These active metabolites form hydrogen bonds with antigen surface molecules, such as peptidoglycans, thereby interfering with pathogen adhesion to surfaces [37, 38], directly eliminating pathogens [39], inhibiting biofilm formation [40], and neutralizing both endo- and exotoxins [41]. Taurolidine in TP remains stabilized by polyvinylpyrrolidone until it is utilized in vivo. TP can be used as both an antiseptic (directly applied to the surgical site) or as a disinfectant (to treat the surface of a medical device). Notably, TP possesses a unique characteristic among antiseptics and disinfectants—it can be safely exposed to the bloodstream [42].

The objective of this study is to estimate the infection rate of CIED following intra-procedural use of TP and to contribute to the safety assessment of TP under standard conditions.

## Methods

### Study enrolment

Patients gave informed consent freely before enrollment into the study.

All consecutive CIED procedures including de novo implantation, pulse generator replacement, lead revision, upgrade or downgrade covered with TP at the participating centers between 01/01/2020 and 30/11/2022 were included (no leadless PM; no ILR).

The original study design allowed variability of TP use across centers, reflecting local practices and preferences, which led to some differences in application. Depending on study site, TP was either used in all procedures (n = 3 centers) or only those deemed at high risk of infection defined as PADIT score > 6 (in 1 center) [9]. All patients whose procedures are covered with TP were followed up for 12 months after discharge from hospital under the ETPR with no exclusion. For a patient undergoing more than one procedure, the follow-up of first CIED procedure employing TP was considered censored in the statistical sense, and the patient was re-entered as a new data unit at the time of the revision procedure, provided TP was used.

### Risk factors

Data on recognized risk factors for CIED infection were collected prospectively [3–9]. Patient related risk factors included the male gender, age < 65 years, acute renal failure (estimated glomerular filtration rate eGFR < 30 ml/min), chronic renal insufficiency (eGFR < 60 ml/min), dialysis dependency (eGFR < 15 ml/min), congestive heart failure, diabetes, malignancy, immunosuppression, dual platelet inhibition or oral anticoagulation, pocket hematoma, previous implant (any foreign bodies except dental implants permanently placed inside the human body, e.g. CIED, vascular graft, osteosynthesis, prosthetic joint replacement) infection, malignancy, chronic obstructive pulmonary disease (COPD), and chronic skin disorder. The procedural and device related risk factors included complex devices (CIED other than single and dual chamber pacemakers and event recorders) for de novo implantation, revision procedures (pulse generator replacement, lead insertion/removal, system upgrade or downgrade and early revision), placement of > 2 leads, abandoned leads in situ, inexperienced operator, procedure duration (skin-to-skin time) ≧ 60 min and prior temporary pacing.

### Procedural details

All CIED procedures were performed by interventional cardiologists, electrophysiologists or cardiothoracic surgeons in a cardiac catheterization laboratory or an electrophysiology laboratory. Operators who performed < 50 CIED procedures per year or < 200 CIED procedures in the preceding 3 years were deemed to be an inexperienced operator, a procedural risk factor (i.e. performing 60 procedures per year in three consecutive years would still be considered a procedural risk factor) [23, 43].

The pre-operative preparation was in accordance with guidelines [23, 24]. A single dose of intravenous antibiotic was administered within 60 min before skin incision, if a cephalosporin was employed. Vancomycin was employed alternatively, which was administered 120 min before skin incision. Chest hair was removed with an electrical clipper. Self-adhesive defibrillator patches and electrocardiogram electrodes were applied to the torso and limbs. The surgical site and adjacent region (the chest wall, upper arms and neck) was cleaned with chlorhexidine 2% gluconate in 70% ethanol. The non-operative area was covered with single use self-adhesive non-woven fabric drapes. Operators wore single use gowns and double gloves. Subcutaneous implantable cardioverter defibrillator (S-ICD) procedures were performed without general anesthetic support at all centers.

All CIED procedures, including de novo implantation, pulse generator replacement, revision, upgrade, downgrade, lead insertion or removal, and S-ICD procedures, were performed using TP cover as per ETPR Standard Operating Procedure (SOP placement, revision and S-ICD from www.etpr.eu and online appendix). A step-by-step description can also be found in the procedure section of the protocol publication by Vonthein et al. No intra-procedural antibiotics, antiseptics, disinfectants, CIED wash, surgical site irrigation, antibiotic collagen sponges, extracellular matrices, or antibiotic-eluting envelopes were used; TP was the sole intervention. No intravenous or oral incremental antibiotic regimen was employed pre- or intra-procedurally. Wound closure was achieved with absorbable sutures for the subcutaneous layer and non-absorbable sutures for the skin. The closed wound was re-cleaned with chlorhexidine 2% gluconate in 70% ethanol and covered with a sterile dressing, with or without a pressure bandage.

### Post-operative care

Postprocedural antibiotic therapy was not administered. Patients were instructed to monitor the surgical site diligently and promptly report any concerns. They were advised to use showering or body wash instead of bathing for a 14-day period and to keep the wound dry during showering, applying a waterproof dressing beforehand. Additionally, they were instructed to refrain from swimming, lifting heavy objects, or engaging in strenuous exercise for 6 weeks.

### Follow-up schedule

Typically, wound inspection occurred two weeks post-surgery, conducted by an attending physician at the implanting center or by a cardiologist in the outpatient setting. Subsequent patient evaluations took place at 3, 6, and 12 months during the initial year and every 6 months thereafter. At each follow-up visit, pertinent endpoint-related data were recorded on the corresponding case report form and reviewed by the endpoint committee for adjudication as necessary.

### Endpoints

The primary endpoint was defined as any “major” CIED infection [23] that resulted in:

1. CIED system removal;
2. an invasive procedure (i.e. pocket revision without removal);
3. pocket revision or CIED system removal after systemic antibiotic therapy;
4. chronic antibiotic therapy without an invasive procedure;
5. death.

The secondary endpoints encompassed all-cause mortality, major CIED infection, minor CIED infection (defined as superficial wound infection without penetration into the CIED pocket, such as wound dehiscence), adverse events attributable to TP, and complications. These complications included pain, arrhythmia, hematoma, lead dislodgement, pericardial effusion, pneumothorax, and pulse generator migration, which either resolved spontaneously or necessitated an invasive corrective procedure (such as pocket evacuation, lead repositioning, pericardiocentesis, or chest drain insertion) within 6 weeks following the index procedure. Additionally, the assessment included instances of CIED malfunction, such as premature battery depletion or ineffective/inappropriate therapy, throughout the entire follow-up period.

### Infection ascertainment

CIED infection was diagnosed following established guidelines [23], utilizing clinical history, physical examination, trans-thoracic and/or transesophageal echocardiography, complete blood count, and repeat blood cultures (with at least 3 sets drawn from various peripheral sites excluding central venous access devices). Infections were confirmed if CIED components were exposed. Whenever the device pocket was accessed, swabs and tissue samples were obtained from the hardware and interior of the pocket, which were then submitted for microbiological analysis.

### Institutional oversight

The study was conducted in compliance with the Declaration of Helsinki and received approval from the Ethics Committee of the University of Kiel under Protocol Code Version 420/21 V2.0 (dated November 15, 2022), as well as from the ethical committees of all participating centers.

### Statistical analysis

The primary analyses were centered on the unadjusted event rate (i.e., the proportion of patients experiencing the event among those at risk) within a specified follow-up period, accompanied by estimation of a 95% confidence interval (CI) using binomial statistics. Patients with follow-up durations shorter than the specified period were excluded from the denominator. Secondary analyses employed the Kaplan–Meier method to accommodate deaths and revisions, treating them either as competing risks or censoring events. Adverse events and complications were evaluated using logistic regression, considering each risk factor individually or their cumulative count.

#### Sample size

The WRAP-IT trial reported major CIED infection rates in the control arm of 0.8% and 1.2% at 3 and 12 months respectively [21]. The PADIT trial reports a major CIED infection rate of 1.03% at 12 months [44]. Assuming lower-than-reported event rates of 0.33% and 0.5% at 3 and 12 months respectively (the same ratio as in the WRAP-IT trial), the ETPR needs a sample size > 572 procedures to exclude a difference of 0.47% (= 0.8 − 0.33%) in the 3-month event rate and > 681 procedures to exclude a difference of 0.503% (= 1.03 − 0.5%) with 95% confidence at twelve months.

## Results

### Study enrolment and baseline characteristics

**Fig. 1:**
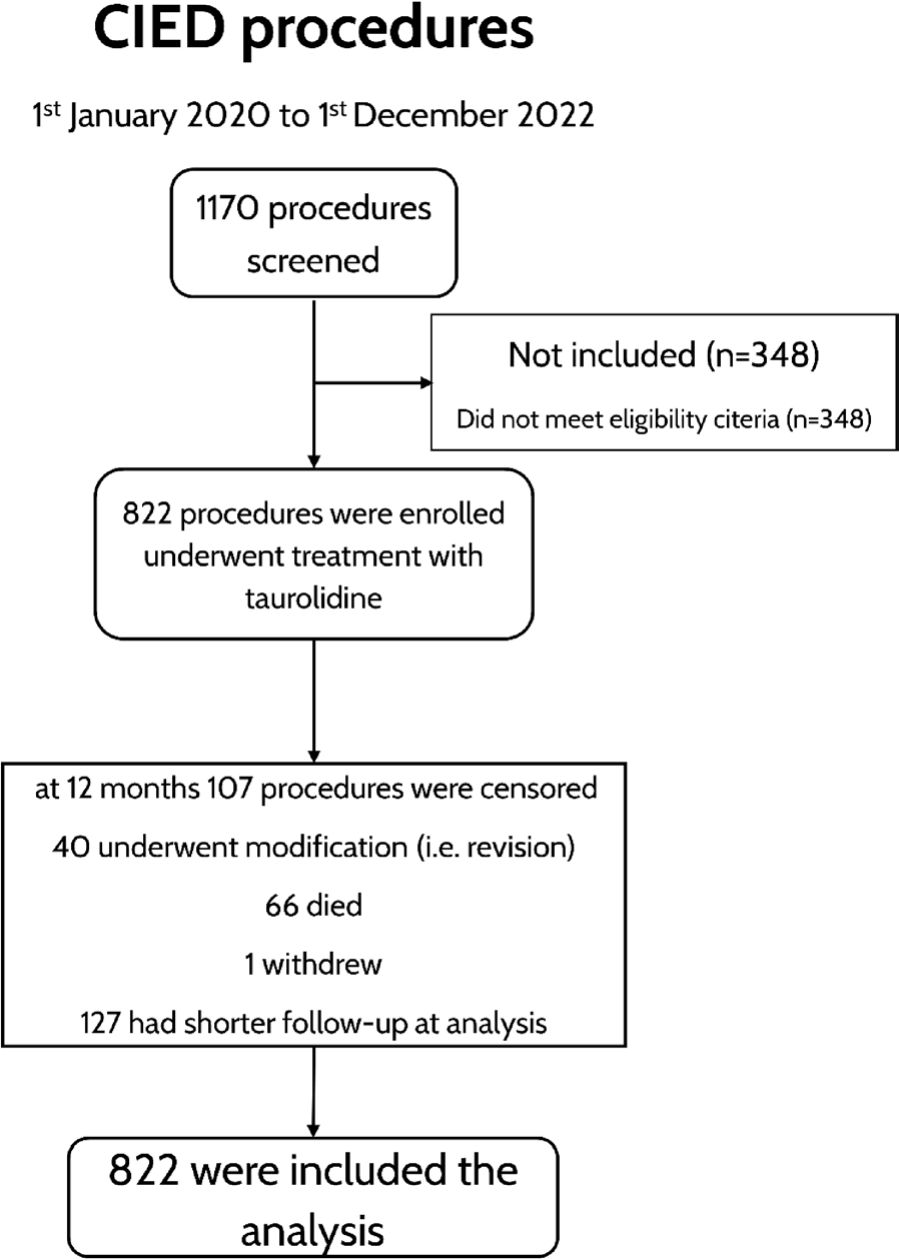
Enrolment and follow-up of the European TauroPace™ Registry. CIED: cardiac implantable electronic device

From January 2020 to November 2022, TP was applied in 822 out of 1170 procedures conducted across the 4 study centers (Fig. 1). One center limited TP usage to procedures deemed high-risk (PADIT score > 6), so that 348 procedures without TP were not recruited. After observing infections in two low-risk patients who did not receive TP, this center transitioned to using TP for all subsequent patients. Our study was specifically designed to assess procedures utilizing TP exclusively, aiming to estimate the infection rate in our standard of care. Furthermore, implementing a clear algorithm (i.e., TP for all patients) ensured adherence to procedural steps, thus minimizing omissions. Patient, procedure, and device characteristics for procedures with complete 12-month follow-up are detailed in Table 1. Figure S1 illustrates the distribution of risk factors for CIED infection within our cohort. Complete data for the entire follow-up duration and for the 3-month follow-up are provided in Tables S1 and S2 in the online appendix.

**Table 1:** Risk factors for CIED infection in 588 procedures on 565 patients with complete 12-month follow-up

	Number	Median	Quartiles
Age (y)	588	76	67; 82
BMI	588	27.1	24; 30
Duration (min)	588	41	30; 52
Risk factors	588	5	3; 6
Patient risk factors	588	3	2; 5

	Number	%	95% CI
Male	395	67.2	63.2 to 71
Age < 65	128	21.8	18.5 to 25.3
Acute renal failure	61	10.4	8.03 to 13.1
Chronic renal insufficiency	243	41.3	37.3 to 45.4
Dialysis dependence	17	2.89	1.69 to 4.59
Congestive heart failure	352	59.9	55.8 to 63.9
Diabetes	196	33.3	29.5 to 37.3
Immunosuppression	68	11.6	9.09 to 14.4
Oral anticoagulation/DPI	383	65.1	61.1 to 69
Pocket hematoma	30	5.1	3.47 to 7.2
Previous implant infection	7	1.19	0.48 to 2.44
Malignancy	107	18.2	15.2 to 21.6
COPD	54	9.18	6.97 to 11.8
Chronic skin disorders	45	7.65	5.64 to 10.1

	Number	Median	Quartiles
Procedure & CIED risk factors	588	1	0; 2

	Number	%	95% CI
ICD, CRT-P, CRT-D, or S-ICD de novo	130	22.1	18.8 to 25.7
Not de novo	200	34	30.2 to 38
Leads > 2	54	9.18	6.97 to 11.8
Abandoned leads in situ	46	7.82	5.78 to 10.3
Inexperienced operator	150	25.5	22 to 29.2
Duration > 59 min	116	19.7	16.6 to 23.2
Temporary pacing	22	3.74	2.36 to 5.61
Device			
PM	321	54.6	50.5 to 58.7
ICD	99	16.8	13.9 to 20.1
CRT-P	42	7.14	5.2 to 9.53
CRT-D	119	20.2	17.1 to 23.7
S-ICD	7	1.19	0.48 to 2.44
CCM	0	0	0 to 0.625
Event recorder	0	0	0 to 0.625
Procedure			
New implantation	388	66	62 to 69.8
Downgrade	9	1.53	0.702 to 2.89
Upgrade	54	9.18	6.97 to 11.8
Change	106	18	15 to 21.4
Revision	31	5.27	3.61 to 7.4
Leads abandoned			
0	542	92.2	89.7 to 94.2
1	40	6.8	4.9 to 9.15
2	6	1.02	0.375 to 2.21
Leads implanted			
0	123	20.9	17.7 to 24.4
1	158	26.9	23.3 to 30.6
2	253	43	39 to 47.1
3	54	9.18	6.97 to 11.8
Early reintervention (i.e., lead dislodgement or placement failure requiring re-intervention**)**	23	3.91	2.5 to 5.81

CIED denotes cardiac implantable electronic device; BMI denotes body mass index; COPD denotes chronic obstructive pulmonary disease; DPI denotes dual platelet inhibition; PPM denotes permanent pacemaker, ICD denotes implantable cardioverter defibrillator; CRT-P denotes permanent pacemaker able to deliver cardiac resynchronization therapy; CRT-D denotes implantable cardioverter defibrillator able to deliver cardiac resynchronization therapy; S-ICD denotes subcutaneous implantable cardioverter defibrillator (a system where all the hardware is implanted subcutaneous, has no transvenous proportion of its lead; CCM denotes cardiac contractility modulation

### Major CIED infection

**Fig. 2:**
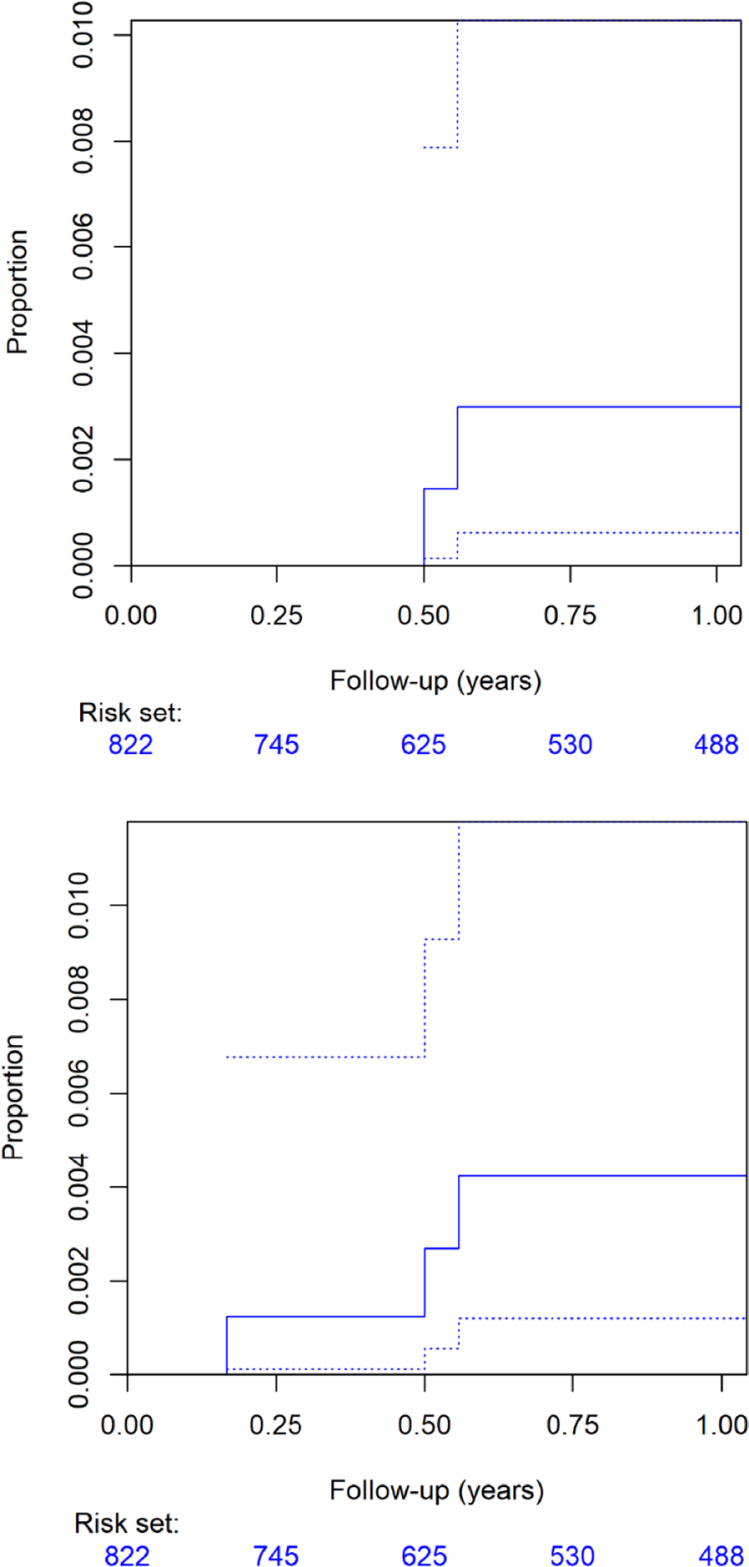
Cumulative incidence curves for local pocket (top) and major (bottom) CIED infection within 1 year post index procedure

In patients who completed the 3-month follow-up after the index procedure, no CIED pocket infections were observed (0%, 95% CI 0–0.46%, Table S3, Fig. 2 top), and one case of CIED-related infective endocarditis was reported (0.125%, 95% CI 0.003–0.70%, Table S3, Fig. 2 bottom) out of 799 CIED procedures performed on 764 distinct patients.

In those who completed the 12-month follow-up, two additional local pocket CIED infections were observed (0.34%, 95% CI 0.04–1.22%, Table 2, Fig. 2 top), resulting in a total of three major CIED infections (0.51%, 95% CI 0.11–1.48%, Table 2, Fig. 2 bottom) out of 588 procedures performed on 565 distinct patients.

**Table 2:** Primary and secondary (sensitivity) estimates of major CIED infection rate in 588 procedures on 565 patients with complete 12-month follow-up

Event	n	Population	Rate (%)	95%-confidence interval (%)
Major CIED infection	3	12-month follow-up	0.51	0.1–1.5
		Death or revision = competing risk	0.42	0.12–1.2
		Death or revision = censoring	0.50	0–1.0
Major CIED pocket infection	2	12-month follow-up	0.34	0.04–1.2
		Death or revision = competing risk	0.30	0.06–1.0
		Death or revision = censoring	0.33	0–0.79
Death	66	12-month follow-up	11.2	8.8–14.1
		Revision = competing risk	9.2	7.2–11.5
		Revision = censoring	9.53	7.28–11.7

CIED denotes cardiac implantable electronic device

Including death or revision procedures as competing risks or censoring events altered the 3-month major CIED infection rate to 0.124% (95% CI 0–0.68%, Table S3) and 0.1% (95% CI 0–0.39%, Table S3), respectively. Similarly, considering these events competing risks changed the 12-month major CIED infection rate to 0.42% (95% CI 0.12–0.68%, Table 2) and 0.50% (95% CI 0–1%, Table 2), respectively.

### Death

**Fig. 3:**
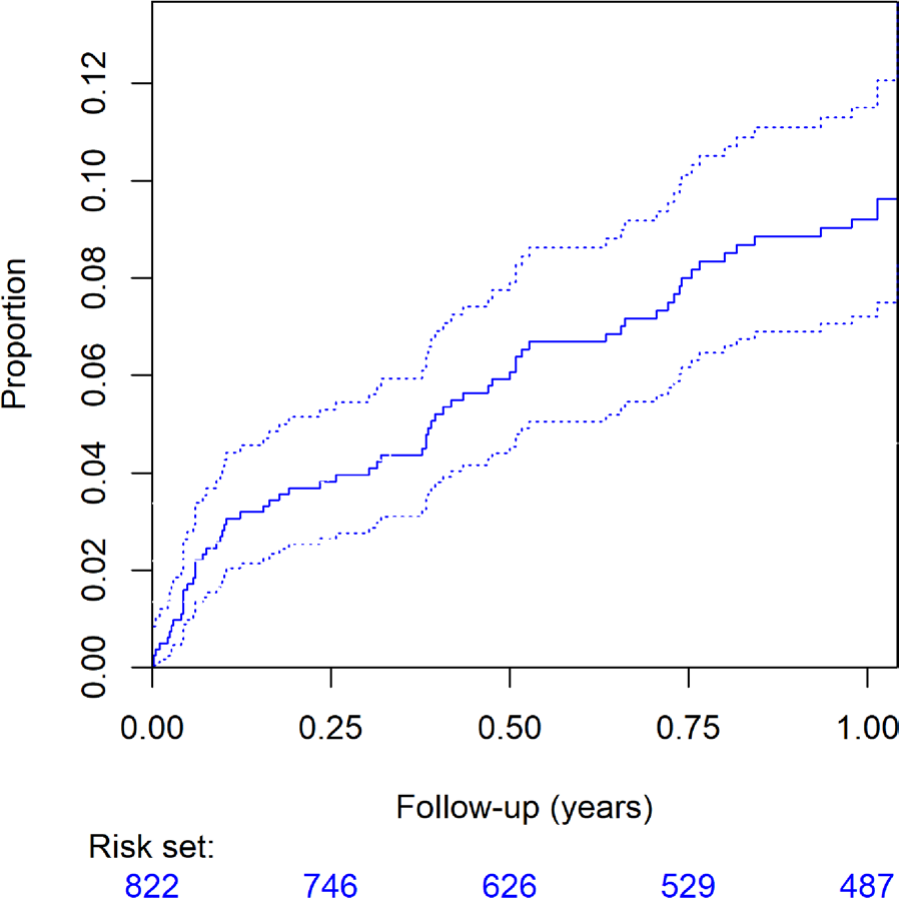
Kaplan Meier survival curve (solid line) within the first year after the index procedure (95% confidence interval in dashed lines)

The 3-month and 12-month all-cause mortality rates were 3.9% (95% CI 2.6–5.5%, Table S3) and 11.2% (95% CI 8.8–14.1%, Table 2, Fig. 3), respectively. Considering revision procedures as either competing risks or censoring events altered the 3-month mortality rate to 3.8% (95% CI 2.6–5.3%) and 3.91% (95% CI 2.55–5.24%), respectively (Table S3). Similarly, for the 12-month mortality rate, these adjustments changed the figures to 9.2% (95% CI 7.2–11.5%) and 9.53% (95% CI 7.28–11.7%), respectively (Table 2).

### Risk factors for premature death post CIED procedure

The risk of death within 12 months for the entire cohort was primarily influenced by 'patient-related risk factors' [OR 1.735; 95% CI 1.412–2.154], rather than 'procedure- and device-related risk factors' [OR 1.167; 95% CI 0.859–1.559]. Logistic regression analysis identified the following factors with an odds ratio (OR) > 1 for death within 3 months of the index procedure (Table S4): acute renal impairment (OR 4.3), chronic renal impairment (OR 3.8), dialysis dependency (OR 4.7), neoplasia (OR 2.7), advanced age (OR 1.05 per year), and temporary pacing (OR 3.7).

### Complications

Throughout the entire median 12-month follow-up period, complications were defined as adverse events that necessitated revision. There were 36 such complications (4.4%, 95% CI 3.1–6.0%) and 73 adverse events adjudicated to be related to the CIED or procedure (8.9%, 95% CI 7.0–11.0%), with 17 of these events classified as serious (2.1%, 95% CI 1.2–3.3%). No adverse events associated with TP were reported. The most frequent non-fatal adverse events included hematoma (requiring revision in 3 cases), lead dislodgement (requiring revision in 6 cases), intra-procedural cardiac arrest (requiring resuscitation in 3 cases), pneumothorax (requiring chest tube in 1 case), pericardial effusion (requiring pericardiocentesis in 1 case), keloid formation (requiring revision in 2 cases), twiddler syndrome (requiring revision in 1 case), and suture granuloma (no revision required).

## Discussion

### Safety and efficacy of taurolidine in prevention of CIED infection

We observed a low rate of infection over the follow-up period. After 12 months, the incidence of pocket infection was 0.34%, and the incidence of major CIED infection was 0.51%. These rates are below the 0.5–4.2% ranges reported in other studies [8, 10]. The universal use of TP in all study patients is a unique aspect distinguishing the ETPR standard of procedure from contemporary standard care and likely contributed to these low event rates. Adherence to guidelines in the study centers may have also positively influenced outcomes. The observed 3-month and 12-month mortality rates of 3.9% and 11.2%, respectively, are comparable to those reported in other studies [12, 21]. No AE could be attributed to TP use. The ETPR cohort exhibits a high burden of comorbidities, which likely necessitated CIED therapy but also may have limited the extent to which CIED therapy could significantly prolong survival.

CIED procedural-related complications (4.4%), adverse (8.9%) and (2.1%) serious adverse events were comparable to contemporary studies (6.0–9.5%) [10, 12]. Thus TP does not appear to impart additional risk to patients compared to common contemporary standard of care.

### CIED infection and risk of death

The median number of risk factors for CIED infection across all procedures was 5, with an interquartile range of 3–6 (Table S1). Two deaths were attributed to infection: one resulted from endocarditis associated with the CIED (15 preprocedural risk factors), and the other from a pocket infection with positive blood cultures (5 preprocedural risk factors). In contrast, the third patient (2 risk factors), survived, likely due to fewer device-related complications, gender and better management of comorbid conditions. This highlights the impact of device complexity, lead issues, and comorbidities on the severity and outcomes of CIED infections. All three patients had renal impairment as a major risk factor. Given the limited number of endpoint events, multiple regression could not be performed.

### Clinical use of taurolidine

TP is user-friendly and versatile. It can be applied to wipe external hardware surfaces, irrigate surgical sites, and flush the inner lumen of catheters exposed to the bloodstream. A fair amount of clinical experience and evidence support the safety and efficacy of TP in both preventing and treating established CIED and VAD infections across various clinical situations [32, 33, 35, 45]. Taurolidine likely has an impact on reducing the rate of CIED infections, but it should be viewed primarily as an adjunct to a comprehensive infection prevention strategy. The primary determinants of infection control include proper surgical technique, stringent sterile barrier precautions, thorough personnel training, and impeccable hygiene standards. While taurolidine can enhance infection prevention efforts, its effectiveness is maximized only when it complements these fundamental practices. Thus, achieving the lowest possible infection rates relies on the synergistic effect of taurolidine in combination with these essential procedural and environmental controls.

### Cost considerations

The initial cost of TP appears reasonable, particularly when considering potential savings from preventing costly complications like CIED infections. Studies assessing the cost-effectiveness of taurolidine as an antimicrobial agent have consistently shown positive outcomes in different departments [46–49]. Despite the relatively low cost of TP itself, economic analyses must account for reduced infection-related healthcare costs and enhanced patient outcomes over the long term. Accumulating larger datasets (such as this ongoing registry) through widespread TP use is essential to fully evaluate its potential for cost reduction.

Recent studies, indicate that a significant proportion of CIED infections occur within the first weeks [21, 50]. Our study, conducted across four centers, observed one infection within three months among 822 procedures where TP was employed. This suggests a substantial potential for cost reduction associated with reduced infection rates [17]. Improved infection prevention with TP has the potential to enhance clinical outcomes, lowering morbidity and mortality rates in patients undergoing CIED procedures. This not only benefits individual patients but also enhances overall healthcare quality and resource utilization. While TP shows promise in infection reduction, clinicians must consider patient-specific variables, procedural risks, and institutional protocols when deciding its use.

### Binomial and Kaplan–Meier statistics

The ETPR departs from randomized clinical trials or other observational registries in using binomial statistics as the primary analyses and the conventional Kaplan–Meier method only as the secondary analyses. The exact binomial confidence interval reliably maintains the specified degree of confidence conservatively for regulatory conclusions drawn from this study, even in the presence of rare events. We present two Kaplan–Meier estimates that differ in considering death and revision as either censored times or competing risks. These address the estimands needed for different purposes and may thus seem more interesting depending on the task at hand.

### Limitations

This is a non-randomized prospective cohort and our data therefore do not meet the same level of evidence as a randomized controlled trial. Results observed in clinical studies might not be realized in clinical practice when an intervention is rolled out to the real world. Operators’ experience and proficiency and center volume and protocols are likely to be major determinants on the safety and efficacy of any form of treatment. The patient cohort and their CIED procedures were representative of the study centers (i.e., no selection or selection based solely on infection risk); however, these cohorts may not be indicative of those at other centers.

Another notable limitation identified is the absence of a control group in our study. A control group functions as a reference against which the treatment group (patients receiving TP) can be assessed. Typically, the control group would receive either standard treatment or a placebo, facilitating an accurate evaluation of TP's actual impact on reducing infection rates compared to not using TP. Despite employing various statistical adjustments to align our findings with those of RCTs, the absence of a control group makes it difficult to attribute the observed outcomes solely to the effects of TP. Put simply, while we compare our results with those reported in other studies, this does not obviate the necessity of an internal control group within our study design. A prospectively observed control group would need to consist of procedures from other sites, as the participating sites consistently chose to use TP in all cases.

Lacking subgroup analyses in the context of the primary endpoint may represent another limitation. However, as this study represents the initial findings from the registry, conducting a preliminary subgroup analysis is a prudent approach. Due to the infrequency of infections, we focused on premature death when exploring potential CIED treatment effects within specific subsets of patients (Table S4). This preliminary analysis serves as a foundation for deeper investigations into broader subgroups as more data accumulate over time. This expansion is contingent upon observing a sufficient number of endpoint events over a 36-month follow-up period involving 2300 patients. By waiting for more comprehensive data, we can conduct more robust subgroup analyses that offer deeper insights into how TP may affect the occurrence of CIED infection in different patient groups over extended periods.

## Conclusions

The adjunctive use of a taurolidine containing solution to cover consecutive or intentionally selected high risk patients undergoing all forms of CIED procedures was associated with a low rate of CIED infection after 3 and 12 months of follow-up. No adverse events attributed to TP were observed.

## Supplementary Information

Additional file 1: Supplementary Tables and Figure.

Additional file 2: Standard Operating Procedures.

## Data Availability

Data is provided within the manuscript and supplementary information files. Full data set is available at zenodo.org 10.5281/zenodo.12782138
