# Supplementary material for: Taurolidine-containing solution for reducing cardiac implantable electronic device infection-early report from the European TauroPace™ registry
Source: J Cardiothorac Surg. 2024 Oct 4;19:592. doi: 10.1186/s13019-024-03059-1 (PMC11451193; doi:10.1186/s13019-024-03059-1)

APPENDIX:

**Table S 1**: Prognostic factors for CIED infection in 822 cases in 785 patients. N = sample size, n = cases with feature.

|  | **N** | **Median** | **Quartiles** |
| --- | --- | --- | --- |
| **Age (y)** | 822 | 77 | 67; 82 |
| **BMI** | 822 | 27.2 | 24; 30 |
| **Duration (min)** | 822 | 42 | 30; 54 |
| **Risk factors** | 822 | 5 | 3; 6 |
| **Host risk factors** | 822 | 3 | 2; 4 |
|  | **n** | **%** | **95%CI** |
| **Male** | 549 | 66.8 | 63.5 to 70 |
| **Age < 65** | 172 | 20.9 | 18.2 to 23.9 |
| **Acute renal failure** | 91 | 11.1 | 9.01 to 13.4 |
| **Chronic renal insufficiency** | 360 | 43.8 | 40.4 to 47.3 |
| **Chronic skin disorders** | 56 | 6.81 | 5.19 to 8.76 |
| **Congestive heart failure** | 475 | 57.8 | 54.3 to 61.2 |
| **COPD** | 78 | 9.49 | 7.57 to 11.7 |
| **Diabetes** | 270 | 32.8 | 29.6 to 36.2 |
| **Dialysis dependent** | 21 | 2.55 | 1.59 to 3.88 |
| **Immunosuppression** | 93 | 11.3 | 9.23 to 13.7 |
| **Oral anticoagulation** | 523 | 63.6 | 60.2 to 66.9 |
| **Malignancy** | 148 | 18 | 15.4 to 20.8 |
| **Pocket hematoma** | 33 | 4.01 | 2.78 to 5.59 |
| **Previous implant infection** | 9 | 1.09 | 0.502 to 2.07 |
|  | **N** | **Median** | **Quartiles** |
| **Procedure & CIED risk factors** | 822 | 1 | 0; 2 |
|  | **n** | **%** | **95%CI** |
| **ICD, CRT-P, CRT-D, or S-ICD *de novo*** | 185 | 22.5 | 19.7 to 25.5 |
| **Not *de novo*** | 280 | 34.1 | 30.8 to 37.4 |
| **Leads > 2** | 68 | 8.27 | 6.48 to 10.4 |
| **Leads in (abandoned)** | 60 | 7.3 | 5.62 to 9.3 |
| **Inexperienced operator** | 219 | 26.6 | 23.6 to 29.8 |
| **Temporary pacing** | 34 | 4.14 | 2.88 to 5.73 |
| **Duration > 59 min** | 167 | 20.3 | 17.6 to 23.2 |
| **Device** |  |  |  |
| **PM** | 452 | 55 | 51.5 to 58.4 |
| **ICD** | 140 | 17 | 14.5 to 19.8 |
| **CRT-P** | 64 | 7.79 | 6.05 to 9.83 |
| **CRT-D** | 148 | 18 | 15.4 to 20.8 |
| **S-ICD** | 16 | 1.95 | 1.12 to 3.14 |
| **CCM** | 2 | 0.243 | 0.0295 to 0.876 |
| **Eventrecorder** | 0 | 0 | 0 to 0.448 |
| **Procedure** |  |  |  |
| **New implantation** | 542 | 65.9 | 62.6 to 69.2 |
| **Downgrade** | 10 | 1.22 | 0.585 to 2.23 |
| **Upgrade** | 68 | 8.27 | 6.48 to 10.4 |
| **Change** | 152 | 18.5 | 15.9 to 21.3 |
| **Revision** | 50 | 6.08 | 4.55 to 7.94 |
| **Leads abandoned** |  |  |  |
| **0** | 762 | 92.7 | 90.7 to 94.4 |
| **1** | 52 | 6.33 | 4.76 to 8.21 |
| **2** | 8 | 0.973 | 0.421 to 1.91 |
| **Leads implanted** |  |  |  |
| **0** | 178 | 21.7 | 18.9 to 24.6 |
| **1** | 228 | 27.7 | 24.7 to 30.9 |
| **2** | 348 | 42.3 | 38.9 to 45.8 |
| **3** | 68 | 8.27 | 6.48 to 10.4 |
| **Early reintervention (i.e., lead dislodgement or placement failure requiring re-intervention)** | 37 | 4.5 | 3.19 to 6.15 |

CIED denotes cardiac implantable electronic device; BMI denotes body mass index; COPD denotes chronic obstructive pulmonary disease; DPI denotes dual platelet inhibition; PPM denotes permanent pacemaker, ICD denotes implantable cardioverter defibrillator; CRT-P denotes permanent pacemaker able to deliver cardiac resynchronisation therapy; CRT-D denotes implantable cardioverter defibrillator able to deliver cardiac resynchronisation therapy; S-ICD denotes subcutaneous implantable cardioverter defibrillator (a system where all the hardware is implanted subcutaneous, has no transvenous proportion of its lead; CCM denotes cardiac contractility modulation

**Table S 2**: Prognostic factors for CIED infection in 799 cases in 764 patients with at least 3 months of follow-up. N = sample size, n = cases with feature.

|  | **N** | **Median** | **Quartiles** |
| --- | --- | --- | --- |
| **Age (y)** | 799 | 76 | 67; 82 |
| **BMI** | 799 | 27.2 | 24; 30 |
| **Duration (min)** | 799 | 42 | 30; 52 |
| **Risk factors** | 799 | 5 | 3; 6 |
| **Host risk factors** | 799 | 3 | 2; 4 |
|  | **n** | **%** | **95%CI** |
| **Male** | 535 | 67 | 63.6 to 70.2 |
| **Age < 65** | 168 | 21 | 18.2 to 24 |
| **Acute renal failure** | 85 | 10.6 | 8.59 to 13 |
| **Chronic renal insufficiency** | 344 | 43.1 | 39.6 to 46.6 |
| **Chronic skin disorders** | 56 | 7.01 | 5.34 to 9.01 |
| **Congestive heart failure** | 461 | 57.7 | 54.2 to 61.2 |
| **COPD** | 73 | 9.14 | 7.23 to 11.4 |
| **Diabetes** | 263 | 32.9 | 29.7 to 36.3 |
| **Dialysis dependent** | 21 | 2.63 | 1.63 to 3.99 |
| **Immunosuppression** | 91 | 11.4 | 9.27 to 13.8 |
| **Oral anticoagulation** | 512 | 64.1 | 60.6 to 67.4 |
| **Malignancy** | 143 | 17.9 | 15.3 to 20.7 |
| **Pocket hematoma** | 32 | 4.01 | 2.76 to 5.61 |
| **Previous implant infection** | 9 | 1.13 | 0.516 to 2.13 |
|  | **N** | **Median** | **Quartiles** |
| **Procedure & CIED risk factors** | 799 | 1 | 0; 2 |
|  | **n** | **%** | **95%CI** |
| **ICD, CRT-P, CRT-D, or S-ICD *de novo*** | 182 | 22.8 | 19.9 to 25.8 |
| **Not *de novo*** | 267 | 33.4 | 30.1 to 36.8 |
| **Leads > 2** | 67 | 8.39 | 6.56 to 10.5 |
| **Leads in (abandoned)** | 57 | 7.13 | 5.45 to 9.14 |
| **Inexperienced operator** | 218 | 27.3 | 24.2 to 30.5 |
| **Temporary pacing** | 32 | 4.01 | 2.76 to 5.61 |
| **Duration > 59 min** | 156 | 19.5 | 16.8 to 22.4 |
| **Device** |  |  |  |
| **PM** | 436 | 54.6 | 51 to 58.1 |
| **ICD** | 138 | 17.3 | 14.7 to 20.1 |
| **CRT-P** | 62 | 7.76 | 6 to 9.84 |
| **CRT-D** | 145 | 18.1 | 15.5 to 21 |
| **S-ICD** | 16 | 2 | 1.15 to 3.23 |
| **CCM** | 2 | 0.25 | 0.0303 to 0.901 |
| **Event recorder** | 0 | 0 | 0 to 0.461 |
| **Procedure** |  |  |  |
| **New implantation** | 532 | 66.6 | 63.2 to 69.9 |
| **Downgrade** | 10 | 1.25 | 0.602 to 2.29 |
| **Upgrade** | 66 | 8.26 | 6.45 to 10.4 |
| **Change** | 145 | 18.1 | 15.5 to 21 |
| **Revision** | 46 | 5.76 | 4.25 to 7.6 |
| **Leads abandoned** |  |  |  |
| **0** | 742 | 92.9 | 90.9 to 94.6 |
| **1** | 49 | 6.13 | 4.57 to 8.03 |
| **2** | 8 | 1 | 0.433 to 1.96 |
| **Leads implanted** |  |  |  |
| **0** | 170 | 21.3 | 18.5 to 24.3 |
| **1** | 223 | 27.9 | 24.8 to 31.2 |
| **2** | 339 | 42.4 | 39 to 45.9 |
| **3** | 67 | 8.39 | 6.56 to 10.5 |
| **Early reintervention (i.e., lead dislodgement or placement failure requiring re-intervention)** | 35 | 4.38 | 3.07 to 6.04 |

CIED denotes cardiac implantable electronic device; BMI denotes body mass index; COPD denotes chronic obstructive pulmonary disease; DPI denotes dual platelet inhibition; PPM denotes permanent pacemaker, ICD denotes implantable cardioverter defibrillator; CRT-P denotes permanent pacemaker able to deliver cardiac resynchronisation therapy; CRT-D denotes implantable cardioverter defibrillator able to deliver cardiac resynchronisation therapy; S-ICD denotes subcutaneous implantable cardioverter defibrillator (a system where all the hardware is implanted subcutaneous, has no transvenous proportion of its lead; CCM denotes cardiac contractility modulation

**Table S 3**: Primary, secondary, and sensitivity estimates of 799 CIED procedures conducted in 764 distinct patients and followed-up for three months (91 days)

| **Event** | **n** | **Population** | **Rate (%)** | **95%-confidence interval (%)** |
| --- | --- | --- | --- | --- |
| **Major CIED infection** | 1 | three months follow-up | 0.125 | 0.003 to 0.7 |
|  |  | Death or revision = competing risk | 0.124 | 0.001 to 0.68 |
|  |  | Death or revision = censoring | 0.10 | 0 to 0.39 |
| **Major CIED pocket infection** | 0 | three months follow-up | 0 | 0 to 0.46 |
|  |  | Death or revision = competing risk | 0 | - |
|  |  | Death or revision = censoring | 0 | - |
| **Death** | 31 | three months follow-up | 3.9 | 2.6 to 5.5 |
|  |  | revision = competing risk | 3.8 | 2.6 to 5.3 |
|  |  | revision = censoring | 3.91 | 2.55 to 5.24 |

***Table S4:*** Effects in logistic regression from 3-months-mortality on 24 single variables

|  | **OR.unit** | **95%-conf. int.** | **P value** | ***** |
| --- | --- | --- | --- | --- |
| **Patient risk factors** | **1.657** | **(1.33 to 1.79)** | **1.4e-08** | ***** |
| Acute renal failure | **4.269** | **(2.38 to 7.46)** | **5.7e-07** | ***** |
| Chronic renal insufficiency | **3.761** | **(2.21 to 6.65)** | **2.1e-06** | ***** |
| Neoplasia | **2.705** | **(1.57 to 4.57)** | **0.00024** | ***** |
| Age (years) | **1.05** | **(1.02 to 1.08)** | **0.00031** | ***** |
| Skin disease | **3.392** | **(1.63 to 6.61)** | **0.00055** | ***** |
| Dialysis | **4.686** | **(1.62 to 12)** | **0.002** | ***** |
| Temporary pacing | **3.667** | **(1.5 to 8.12)** | **0.0023** | * |
| Age < 65 | **0.4023** | **(0.165 to 0.838)** | **0.026** |  |
| Anticoagulation | **1.831** | **(1.06 to 3.32)** | **0.037** |  |
| Immunosuppression | **1.953** | **(0.986 to 3.63)** | **0.042** |  |
| COPD | **1.707** | **(0.792 to 3.36)** | **0.14** |  |
| Previous implant infection | **3.181** | **(0.468 to 13.5)** | **0.15** |  |
| Heart failure | **1.408** | **(0.848 to 2.39)** | **0.19** |  |
| Leads>2 | **0.4811** | **(0.115 to 1.34)** | **0.23** |  |
| Diabetes | **1.349** | **(0.805 to 2.23)** | **0.25** |  |
| Pocket hematoma | **1.536** | **(0.446 to 4.05)** | **0.43** |  |
| Male | **0.994** | **(0.595 to 1.7)** | **0.46** |  |
| **Device** | **1.238** | **(0.688 to 2.14)** | **0.53** |  |
| **Procedure & device risk factors** | **1.058** | **(0.834 to 1.33)** | **0.63** |  |
| Inexperienced interventionalist | **1.051** | **(0.591 to 1.8)** | **0.86** |  |
| Generator exchange | **1.035** | **(0.608 to 1.72)** | **0.9** |  |
| **Procedure >59 min** | **0.8122** | **(0.407 to 1.5)** | **0.98** |  |
| Leads abandoned | **0.9915** | **(0.337 to 2.34)** | **0.99** |  |

COPD denotes chronic obstructive pulmonary disease; OR denotes odds ratio, * = statistically significant in a Bonferroni-Holm procedure.

Figure S1: Distribution of risk factors among CIED procedures covered by TP


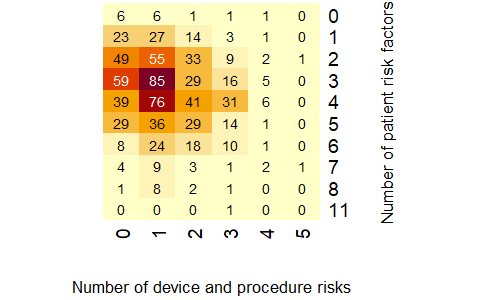

Supplement: Supplementary file 1 — Additional file 1: Supplementary Tables and Figure. [file 13019_2024_3059_MOESM1_ESM.docx]
