# Supplementary material for: Taurolidine-containing solution for reducing cardiac implantable electronic device infection-early report from the European TauroPace™ registry
Source: J Cardiothorac Surg. 2024 Oct 4;19:592. doi: 10.1186/s13019-024-03059-1 (PMC11451193; doi:10.1186/s13019-024-03059-1)
Supplement: Supplementary file 2 — Additional file 2: Standard Operating Procedures. [file 13019_2024_3059_MOESM2_ESM.pdf]

TauroPace

Step by step instructions

PREPARATION:

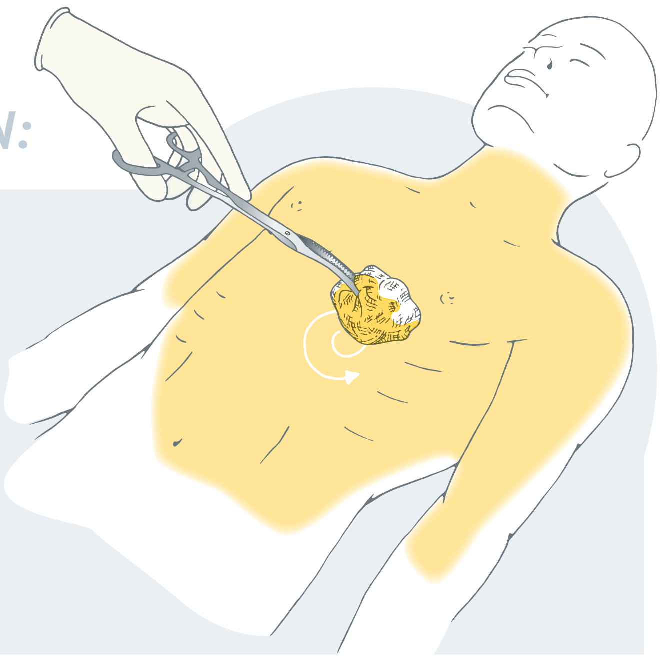

1

Surgical skin preparation must be carried out as close as possible to commencement of the procedure with an antiseptic solution recommended (consider fascial plane block for local anesthesia).

2

Drape the patient.

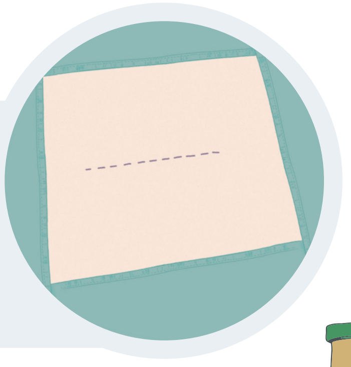

3

Transfer **TauroPace** to a gallipot containing surgical swabs.

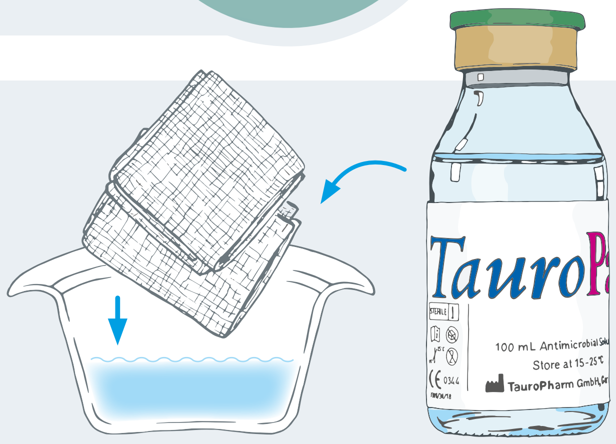

CIED-placement

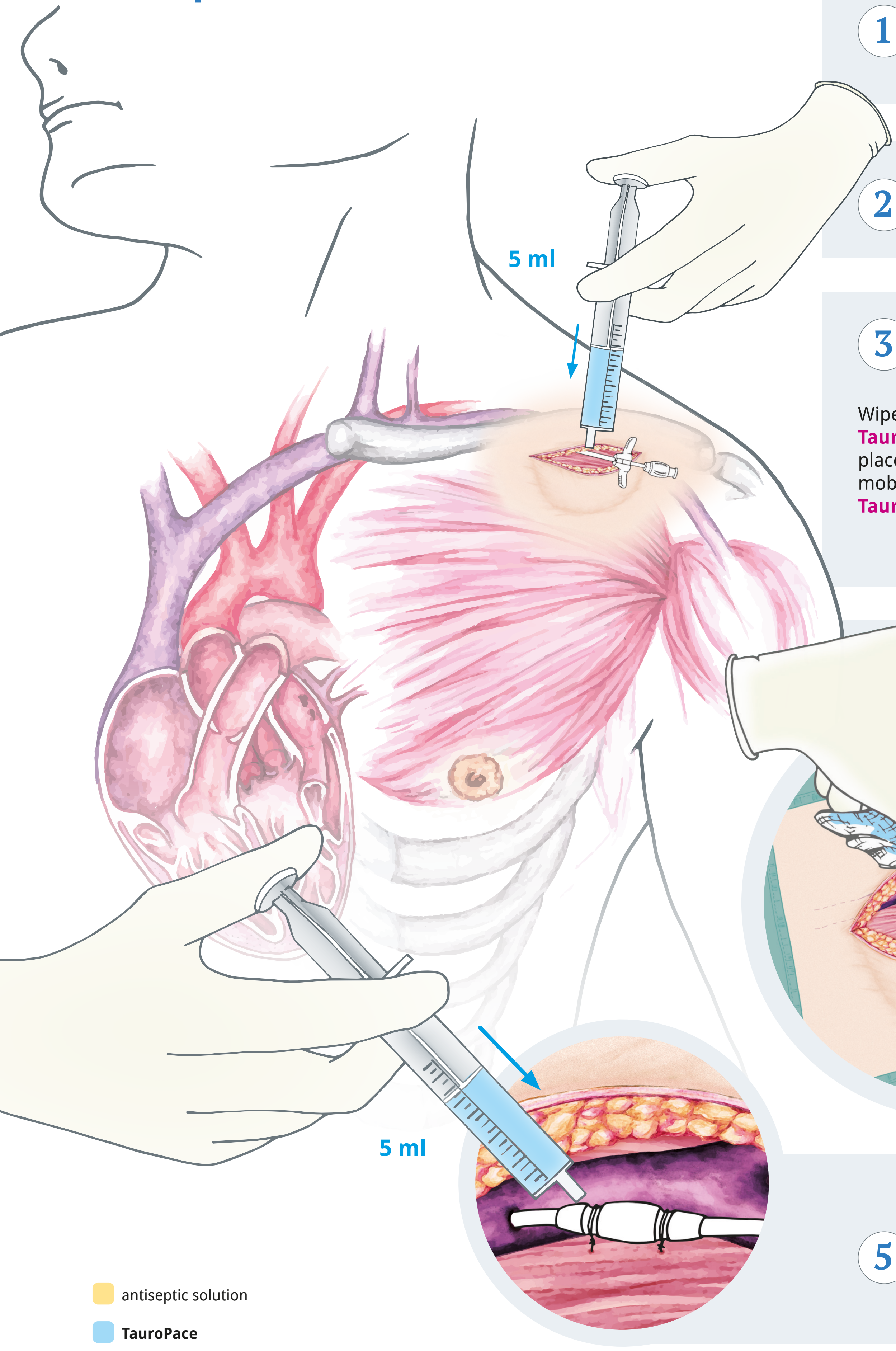

1

After skin incision and gaining access to/preparation of the vein wipe down sheath/dilator with **TauroPace** before placement.

2

After placement rinse the surgical site with **TauroPace**.

3

Wipe down the lead with a **TauroPace** soaked swab prior to placement. Please ensure to mobilise the suture sleeve with a **TauroPace** soaked swab (important).

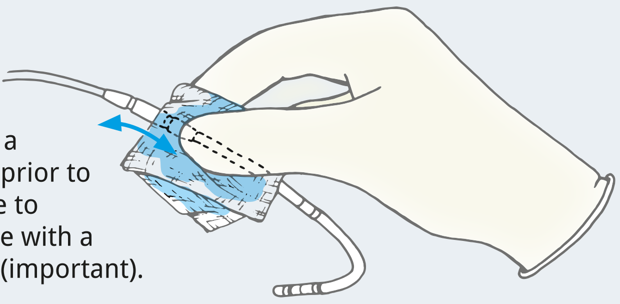

4

Move the suture sleeve in final position with a **TauroPace** soaked swab.

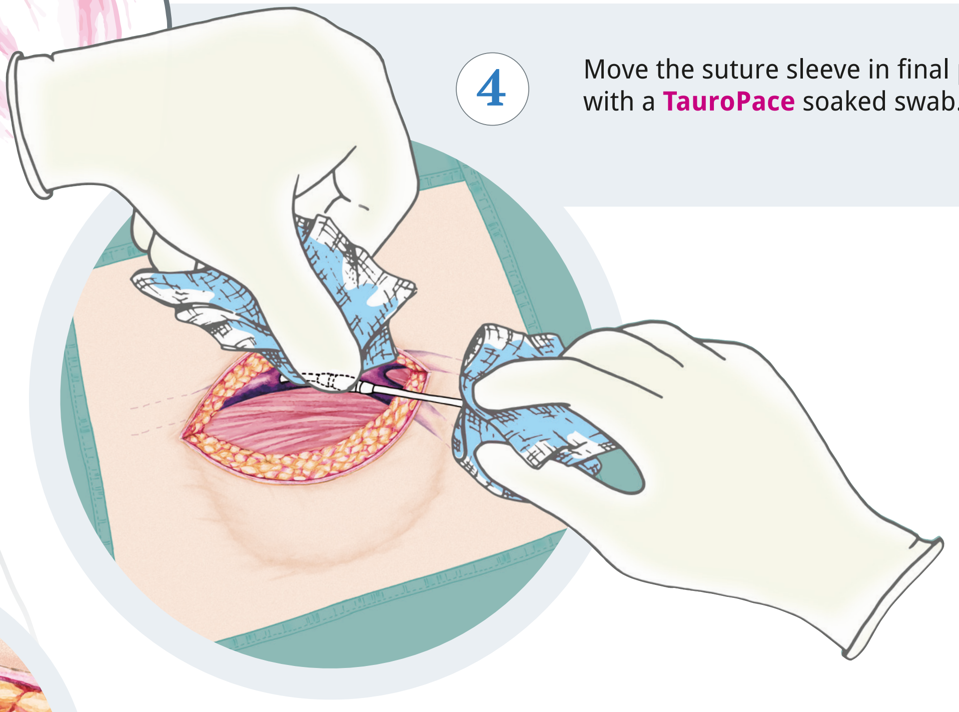

5

After placement of the lead(s), rinse the surgical site with **TauroPace**.

antiseptic solution

TauroPace

Generator substitution or revision

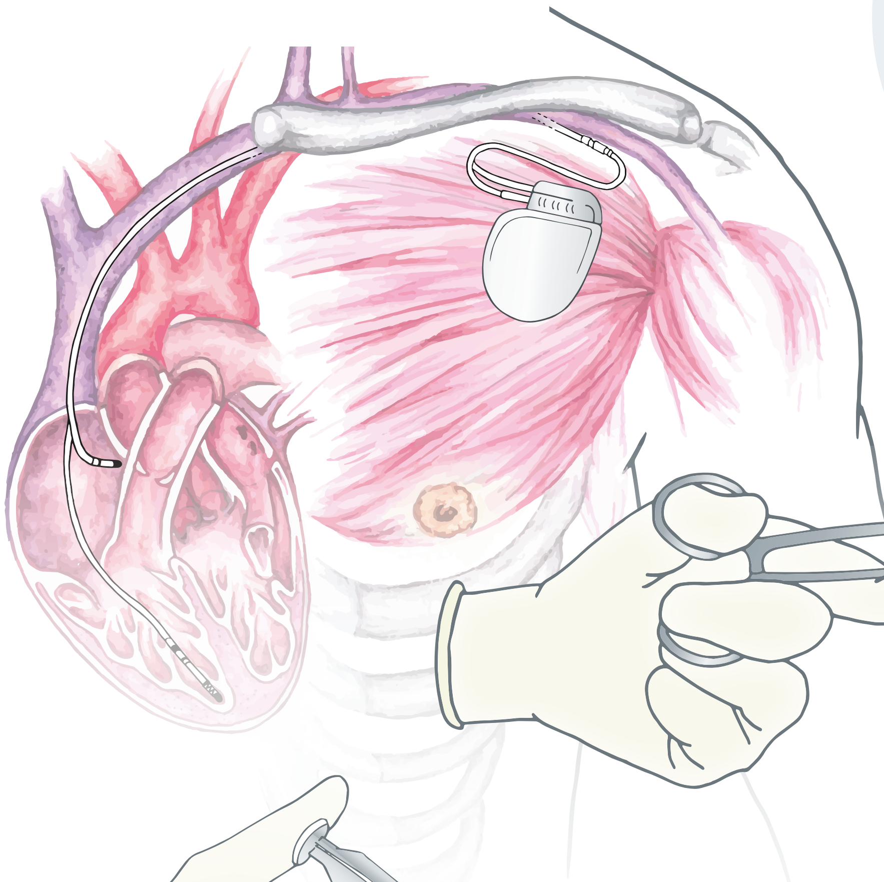

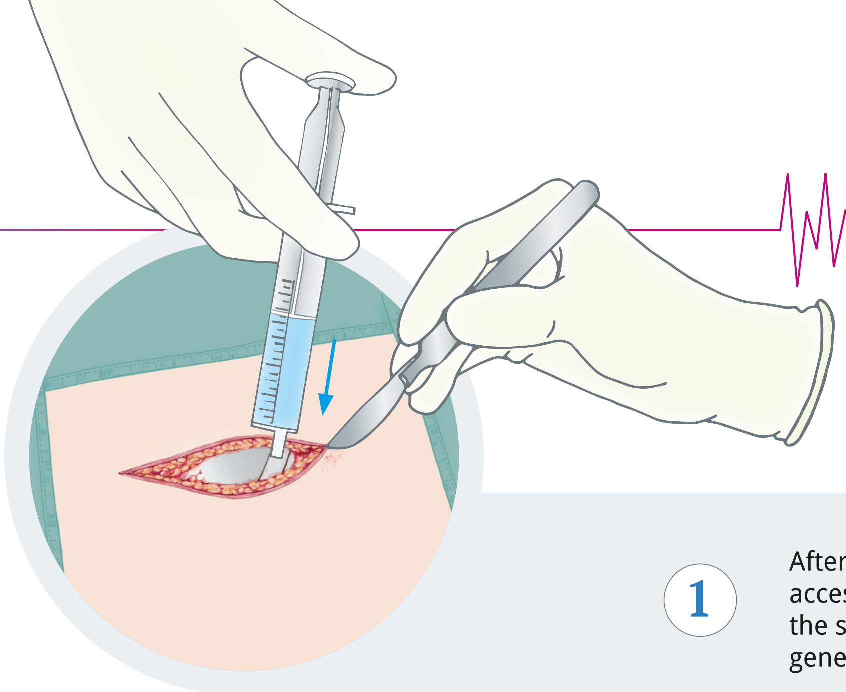

1 After skin incision and gaining access to the pocket, rinse the surgical site with the old generator in place.

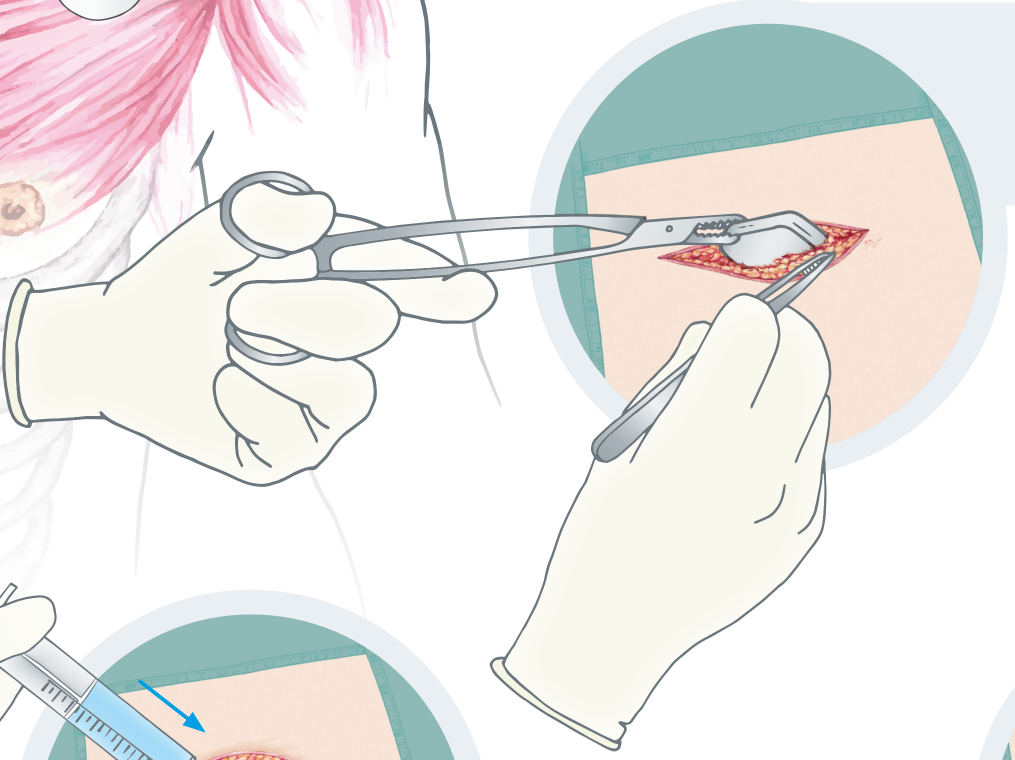

2 Mobilise the old generator.

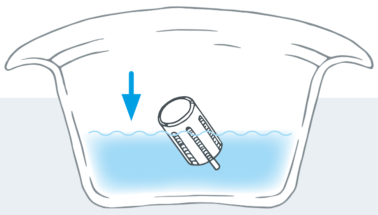

3 Immerge the torque wrench in **TauroPace**.

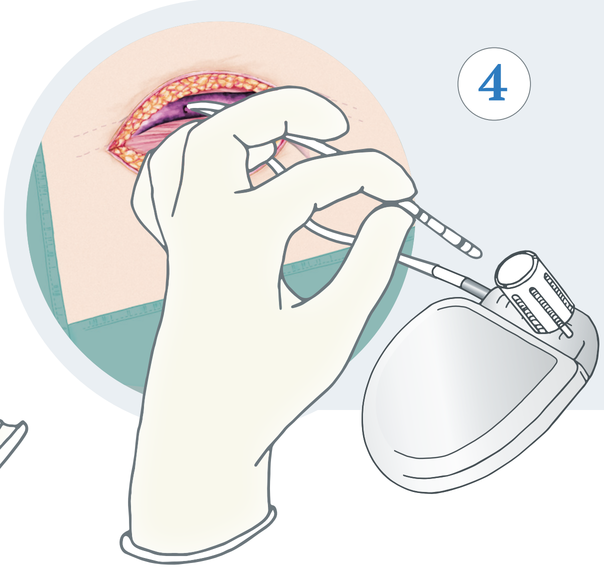

4

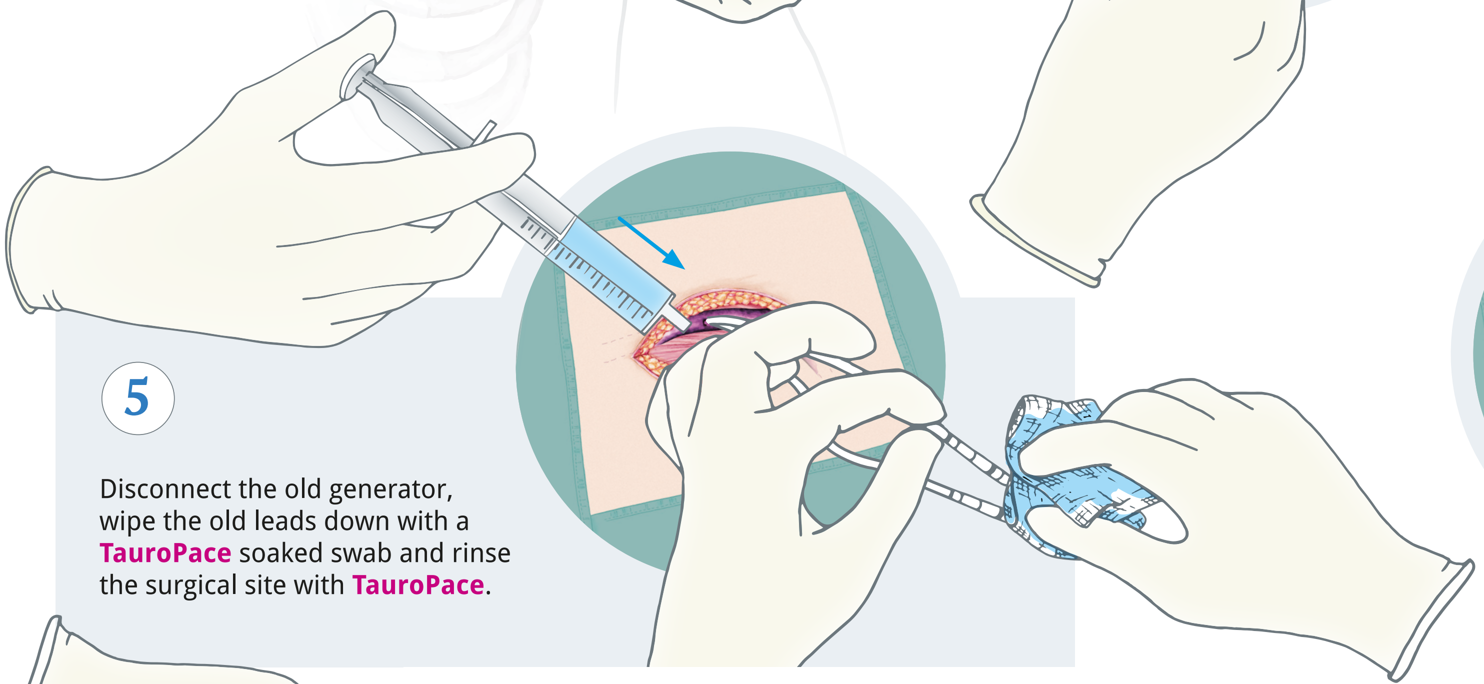

5 Disconnect the old generator, wipe the old leads down with a **TauroPace** soaked swab and rinse the surgical site with **TauroPace**.

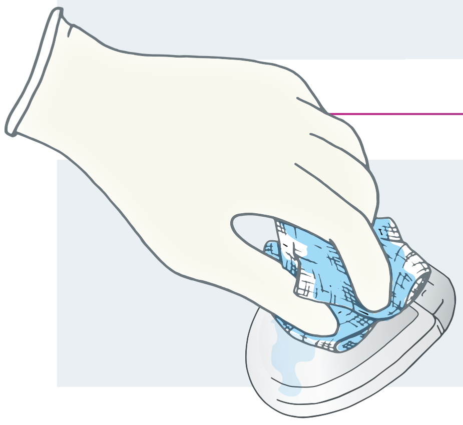

6 Wipe the new generator down with a **TauroPace** soaked swab.

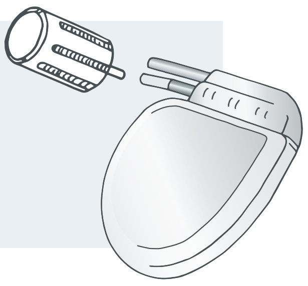

9

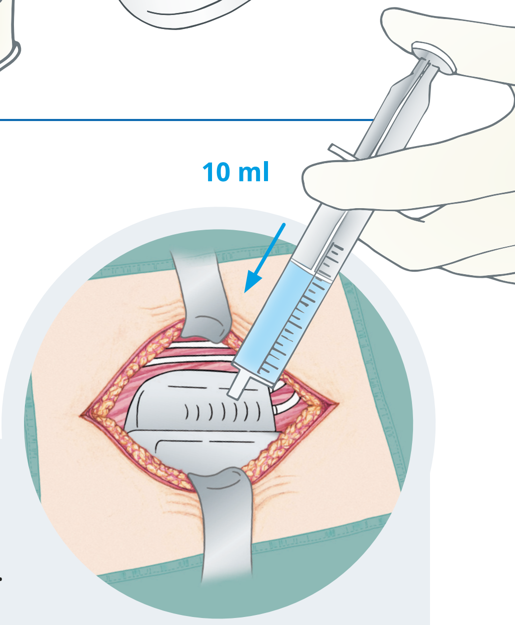

7 Immerge the torque wrench in **TauroPace**.

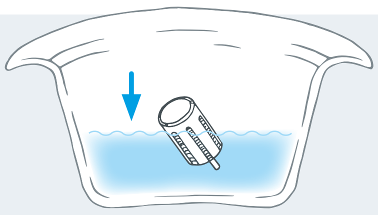

10 After final placement of all hardware, rinse the surgical site with **TauroPace**.

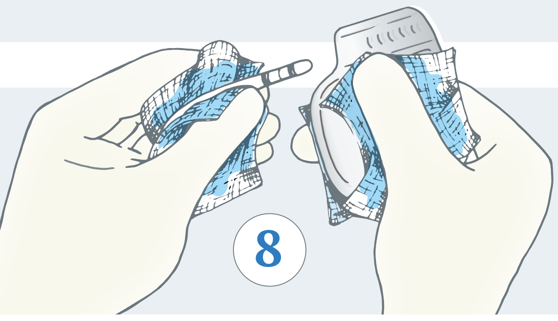

8

Connect the lead(s) to the generator; use **TauroPace** soaked swabs to hold on to the lead and the generator.

11 Remove excess **TauroPace**.

AFTER SKIN CLOSURE:

1 After skin closure wipe down with recommended skin antisepsis.

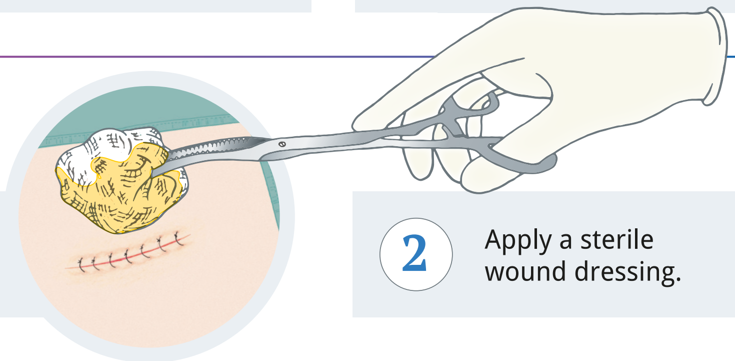

2 Apply a sterile wound dressing.

Use one bottle of **TauroPace** per procedure.

Illustration © Insa Brüning
